# Enhancing the Thermal Stability of Carbon Nanomaterials with DNA

**DOI:** 10.1038/s41598-019-48449-x

**Published:** 2019-08-15

**Authors:** Mohammad Moein Safaee, Mitchell Gravely, Adeline Lamothe, Megan McSweeney, Daniel Roxbury

**Affiliations:** Department of Chemical Engineering, University of Rhode Island, Kingston, Rhode Island 02881 United States

**Keywords:** Carbon nanotubes and fullerenes, Mechanical properties, Organizing materials with DNA, Electronic devices, Mechanical and structural properties and devices

## Abstract

Single-walled carbon nanotubes (SWCNTs) have recently been utilized as fillers that reduce the flammability and enhance the strength and thermal conductivity of material composites. Enhancing the thermal stability of SWCNTs is crucial when these materials are applied to high temperature applications. In many instances, SWCNTs are applied to composites with surface coatings that are toxic to living organisms. Alternatively, single-stranded DNA, a naturally occurring biological polymer, has recently been utilized to form singly-dispersed hybrids with SWCNTs as well as suppress their known toxicological effects. These hybrids have shown unrivaled stabilities in both aqueous suspension or as a dried material. Furthermore, DNA has certain documented flame-retardant effects due to the creation of a protective char upon heating in the presence of oxygen. Herein, using various thermogravimetric analytical techniques, we find that single-stranded DNA has a significant flame-retardant effect on the SWCNTs, and effectively enhances their thermal stability. Hybridization with DNA results in the elevation of the thermal decomposition temperature of purified SWCNTs in excess of 200 °C. We translate this finding to other carbon nanomaterials including multi-walled carbon nanotubes (MWCNTs), reduced graphene oxide (RGO) and fullerene (C_60_), and show similar effects upon complexation with DNA. The rate of thermal decomposition of the SWCNTs was also explored and found to significantly depend upon the sequence of DNA that was used.

## Introduction

Reduced flammability, defined as a high thermal stability and/or low heat release rate, is a desirable feature of novel advanced materials^1–3^. A variety of synthetic flame-retardant chemicals are incorporated into polymer composites^4–6^, and textiles^7–9^, to decrease the flammability of manufactured products such as electronics, building, and construction materials, and furnishings. Synthetic flame retardants can be classified into halogenated^10^, (*e*.*g*. brominated and chlorinated) and non-halogenated^11^ (primarily phosphorus-containing). Studies in laboratory animals and humans have linked the most widely used flame retardants, polybrominated diphenyl ethers (PBDEs), to thyroid disruption^12^, memory and learning problems^13^, and reduced fertility^14^. Unfortunately, the synthetic chemicals added to consumer products to meet federal and state flammability standards have been reported to enter waterways^15^, wildlife^16^, and even human breast milk^17^.

Deoxyribonucleic acid (DNA) has been proven as an intrinsically intumescent flame retardant^18,19^, as it contains the three typical components of an intumescent formulation. First, the phosphate backbone in DNA phosphorylates the substrate and produces a polymeric form of phosphoric acid under flame conditions^18–20^. This acid “char” is thermally-insulating and further protects the substrate surface from oxygen and/or flames. Next, the deoxyribose sugars in DNA tend to dehydrate upon heating, form a char, and release water^18,19^. Finally, the nitrogen-containing nucleobases give rise to the formation of azo-compounds which are able to further induce a char development in addition to the production of non-combustible gases (notably, N_2_, CO_2_ and CO)^18,19^.

Single-walled carbon nanotubes (SWCNTs) functionalized with surfactants^21^, and amphiphilic polymers^22^, have recently been developed and utilized in various disparate fields ranging from targeted anti-cancer drug delivery^23^, to near-infrared optical sensing^24^, and biological imaging^25^. Of significant interest, SWCNTs exhibit flame-retardant properties^26^, extraordinarily high young modulus^27,28^, and thermal conductivity^29^, which make them ideal fillers that can improve both flammability^30^, and mechanical properties of polymer nanocomposites^31,32^. In flame retardant applications, SWCNTs form a structured network that covers the surface of the nanocomposite^26^. Upon exposure to flame at elevated temperature, this layer acts as heat shield to slow the thermal degradation of the polymer below the nanotube film^26^. Enhancing the thermal stability of SWCNTs is therefore desired to decrease the flammability of such composites. Such SWCNTs with enhanced thermal stability will be advantageous in other high temperature applications, *e*.*g*. high temperature sensing^33^, and electronics^34^.

Due to its hydrophobic bases and a hydrophilic backbone, single-stranded DNA has been demonstrated to helically wrap and non-covalently attach to the outside of the SWCNTs by hydrophobic π-π stacking interactions, and enable single-particle dispersion of the SWCNTs in aqueous environments^35^. Probe-tip sonication is generally performed to induce enough energy in order to break up initially bundled SWCNTs and individually disperse them in water^36^. Considering the mentioned flame-retardant ability of DNA coupled with its unique helical conformation on the SWCNTs side-wall, it can act as an effective protection for SWCNTs against thermal decomposition. DNA-SWCNT hybrids are promising candidates to be utilized as flame retardants in the production of flame-resistant nanocomposites due to unique characteristics of SWCNTs and reports on minimal toxicity of DNA-SWCNT hybrids^37,38^, despite the toxicity of convectional flame retardants^12^, raw SWCNTs and surfactant coated SWCNTs^39^.

Thermogravimetric analysis (TGA) can reliably be used to characterize and elucidate the purity of nanomaterials^40^. TGA is a straightforward analytical technique that quantifies mass loss when a material is heated in the absence or presence of oxygen. Information on the thermal decomposition temperature, *i*.*e*. the temperature of maximum weight loss, thermal decomposition rate in a rapid temperature increase^40^, and residual mass of the sample are obtained from the decomposition curve (weight vs temperature or time). The residual mass after decomposition could be due to inorganic nanomaterials, residual metal catalysts from synthesis, or impurities within the sample^41^.

Moreover, confocal Raman microscopy can be utilized in order to characterize the structural integrity of the SWCNTs after exposure to elevated temperatures. The Raman signal from SWCNTs, particularly the intensity of the G-band (located ~1589 cm^−1^), is dependent only on the amount of *sp*^2^-hybridized graphitic carbon present in a sample^42^, and is not affected by noncovalent functionalization. Other signature SWCNT Raman features that can be used to elucidate their structure include the radial breathing mode (RBM, located ~200–300 cm^−1^) and the D-band (~1340 cm^−1^), which correlate to nanotube diameter and disorder of *sp*^2^-hybridization respectively^43^.

In this report, using a thermogravimetric analytical approach, we examined the thermal decomposition characteristics (thermal decomposition temperature and rate) of DNA functionalized carbon nanomaterials. We find that hybridization of the nanomaterials with DNA increased the thermal decomposition temperature of HiPco SWCNTs in excess of 200 °C. We also find that hybridization with DNA significantly increased the thermal decomposition temperature of other carbon nanomaterials such as multi-walled carbon nanotubes (MWCNTs), reduced graphene oxide (RGO), and fullerene (C_60_). Furthermore, the thermal decomposition rate in an instantaneous temperature increase can be controlled by manipulating the sequence of the DNA used in the construction of the hybrids.

## Results and Discussion

**Figure 1:**
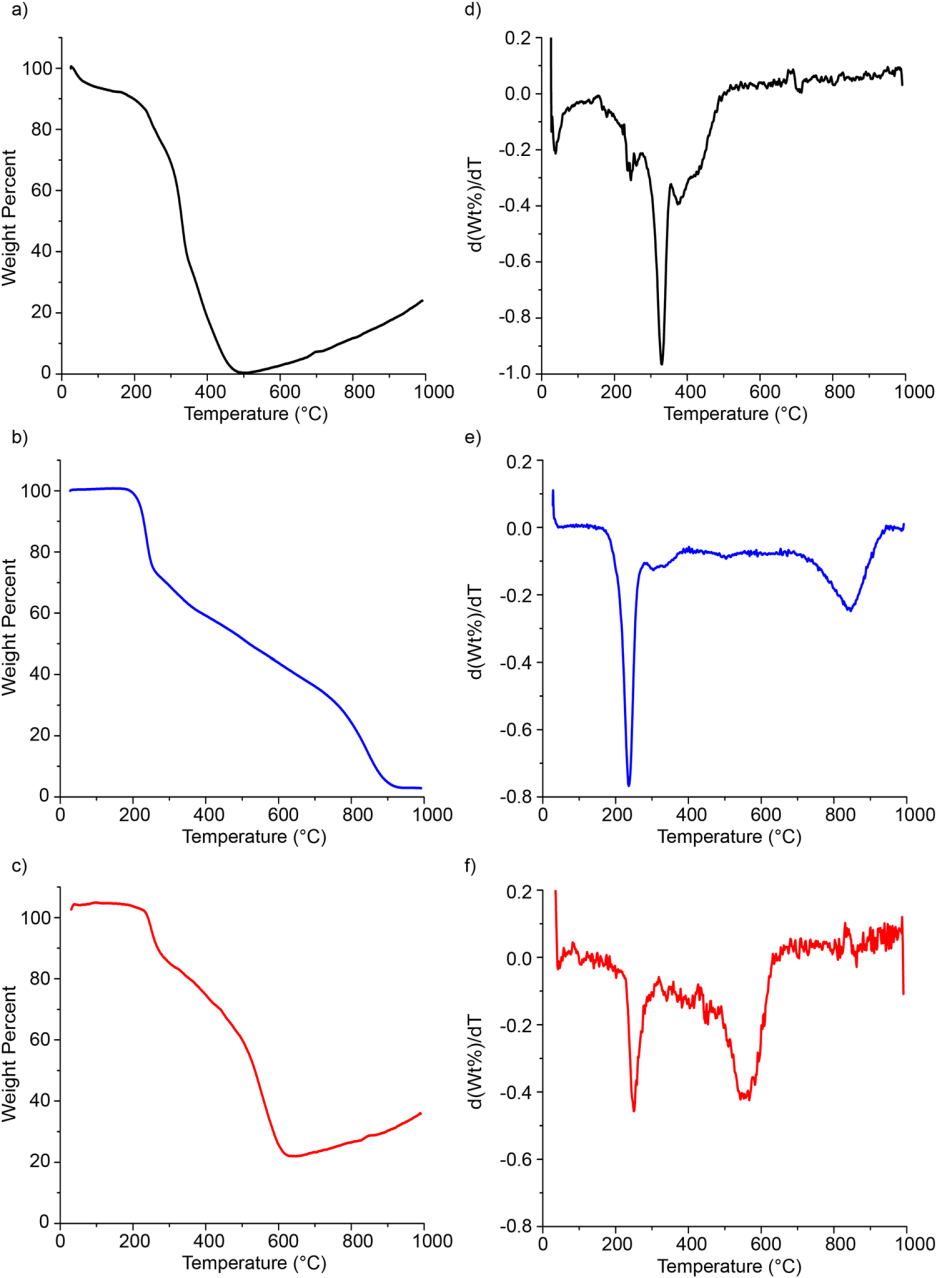
Weight percent versus temperature profiles of (**a**) purified SWCNTs, (**b**) pure (GT)_15_ DNA oligonucleotide, and (**c**) (GT)_15_-SWCNT hybrids. The first-derivatives of these respective plots, d(Wt%)/dT, are computed and presented in (**d**–**f**).

Thermogravimetric analysis was used to assess the thermal decomposition temperature (maximum weight loss temperature) of DNA-SWCNT hybrids in addition to their constitutive components. Weight percent versus temperature data were acquired at the rate of 20 °C/min for raw high-pressure carbon monoxide (HiPco) SWCNTs, purified SWCNTs, pure DNA, and DNA-SWCNT hybrids (Figs 1a–c and S1–S7). In order to delineate the temperature of thermal decomposition, first derivative plots were created (Fig. 1d–f). In the case of raw SWCNTs and purified SWCNTs, an abrupt decrease in the mass of the sample occurred at 311 ± 3 °C and 328 ± 2 °C, respectively (Figs 1a,d and S1a–d). The lower thermal decomposition temperature of the raw SWCNTs compared to the purified SWCNTs was presumably due to the fast oxidization of the iron catalyst particles^44^, which accounted for greater than 40% of the total weight of the raw SWCNT sample, and remained stable past 1000 °C. X-ray photon spectroscopy (XPS) was performed on raw HiPco SWCNTs and the residual material after thermal decomposition to quantify the elemental composition of the sample (Fig. S1e,f). In the raw HiPco sample, only carbon and oxygen were observed as the atomic percentage of iron/iron oxide would be too low in relation to the SWCNTs to detect (Fig. S1e). However, the comparable atomic percentage of the iron to carbon and oxygen in the residual decomposed sample confirms the presence of iron/iron oxide as an oxidizer in the thermal decomposition process (Fig. S1f). Since we purify the DNA-SWCNTs from the catalyst material through ultracentrifugation^35^, purified SWCNTs were used as control throughout the remainder of this study.

Additionally, pure DNA exhibited a continual weight loss over almost the entire examined temperature range. Two temperatures of significant mass loss occurred at 235 ± 1 °C and 838 ± 3 °C (Fig. 1b,e) which were independent of DNA sequence length (Fig. S7). This can be attributed to the fact that DNA starts to decompose near 200 °C, forming significant amounts of char with enhanced thermal stability up to greater than 800 °C^18^.

To interrogate the enhanced thermal stability that DNA imparts on the SWCNTs, the thermal decomposition temperature of SWCNTs functionalized with DNA was compared to that of purified SWCNTs and pure DNA. There were two temperatures of significant weight loss in the DNA-SWCNT decomposition curve (Fig. 1c,f), which could be attributed to the DNA and SWCNT decomposition, respectively. The single-stranded DNA sequence (GT)_15_, when conjugated to SWCNTs, decomposed at 248 ± 2 °C. This represented a marginally elevated decomposition temperature relative to pure (GT)_15_ DNA not conjugated to SWCNTs. This can be attributed to the known flame-retardant characteristics of the SWCNTs^30^. A consistent trend was seen in DNA-SWCNT hybrids constructed from other sequences (Fig. S8a). The second temperature of interest, representing the presumed SWCNT thermal decomposition temperature, was located at 551 ± 3 °C. Compared to the purified SWCNTs, this represented a substantial increase of 250 °C (p < 0.001) which was fairly insensitive to DNA sequence (Fig. S8b). Our working hypothesis is that the DNA, which non-covalently wraps individual SWCNTs, expands upon heating, thus encapsulating and thereby protecting the SWCNT from decomposition. Additionally, we acquired the weight versus temperature profiles for DNA-SWCNTs at the heating rate of 10 °C/min (Fig. S5e,f). Although there is not any substantial change in shape (i.e. appearance of new peaks) in the profile, we observe a split peak for the SWCNT material (major peak at 585 ± 5 °C, minor peak at 524 ± 6 °C), suggesting two populations of SWCNT material. Interestingly, the average of these two peaks results in a value that is not significantly different from the second peak of DNA-SWCNTs at the 20 °C/min heating ramp (554 ± 3 °C) (Fig. S5g).

**Figure 2:**
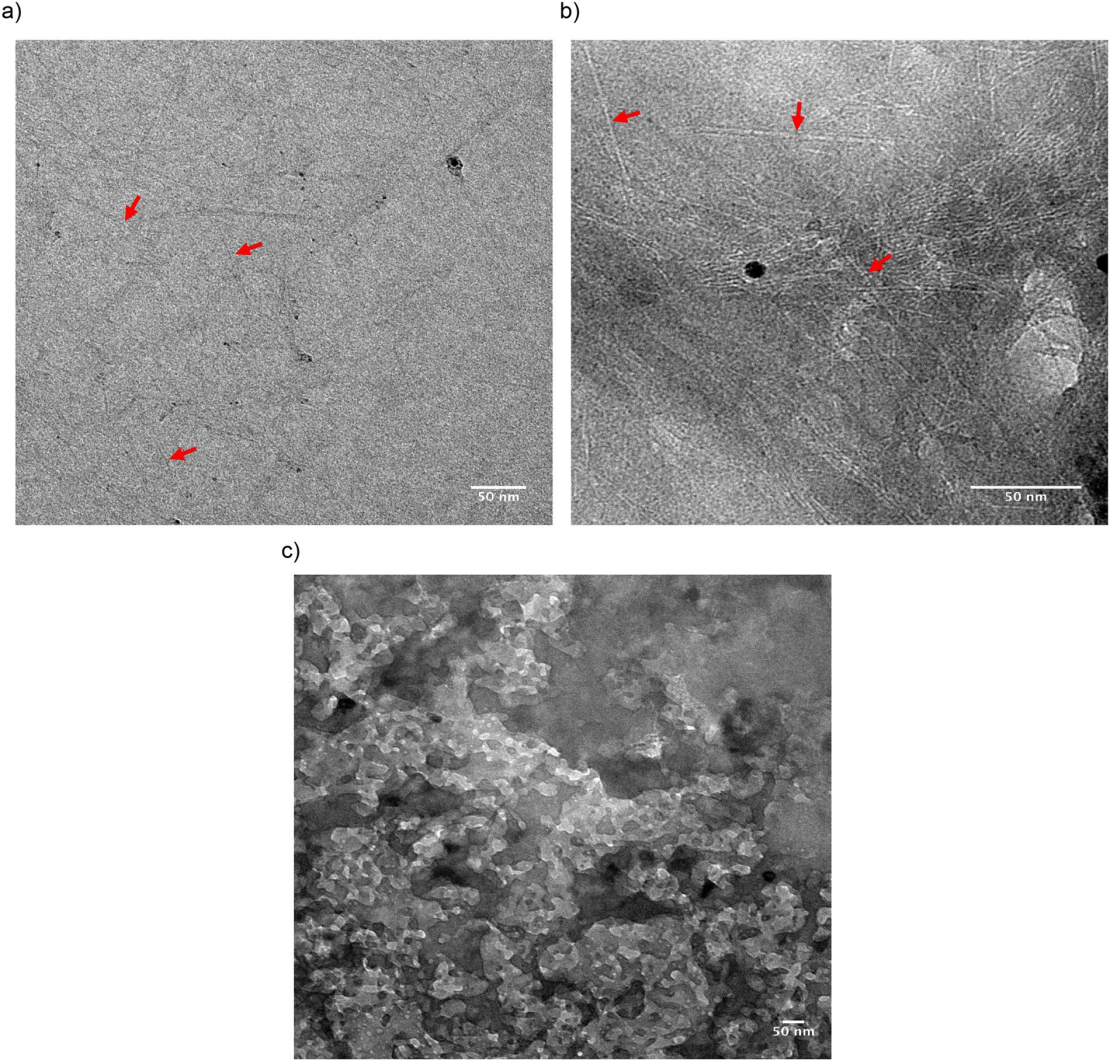
TEM images of (GT)_15_-SWCNTs exposed to (**a**) room temperature, (**b**) 400 °C, or (**c**) 700 °C for 30 minutes. All samples imaged at room temperature. The red arrows point to the SWCNTs.

In order to prove the formation of DNA char on the SWCNTs, transmission electron microscopy (TEM) was performed on (GT)_15_-SWCNT samples exposed to various temperatures. Figure 2a–c illustrate electron micrographs of the dried (GT)_15_-SWCNT hybrids at room temperature, and after exposure to 400 and 700 °C, respectively. Individual SWCNTs are observed in the room temperature sample (red arrows in Fig. 2a), consistent with previous reports^44^. Interestingly, after exposure to 400 °C, SWCNTs appeared embedded in an electron dense structure, (red arrows in Fig. 2b) presumably the char produced by the DNA (Fig. S9). Finally, the image of (GT)_15_-SWCNT hybrids after exposure to 700 °C indicated a different morphology altogether (Fig. 2c), with no resemblance of a tube-like material. We use these TEM images as evidence that a thick char-like layer is forming on the SWCNTs and increasing their thermal stability.

**Figure 3:**
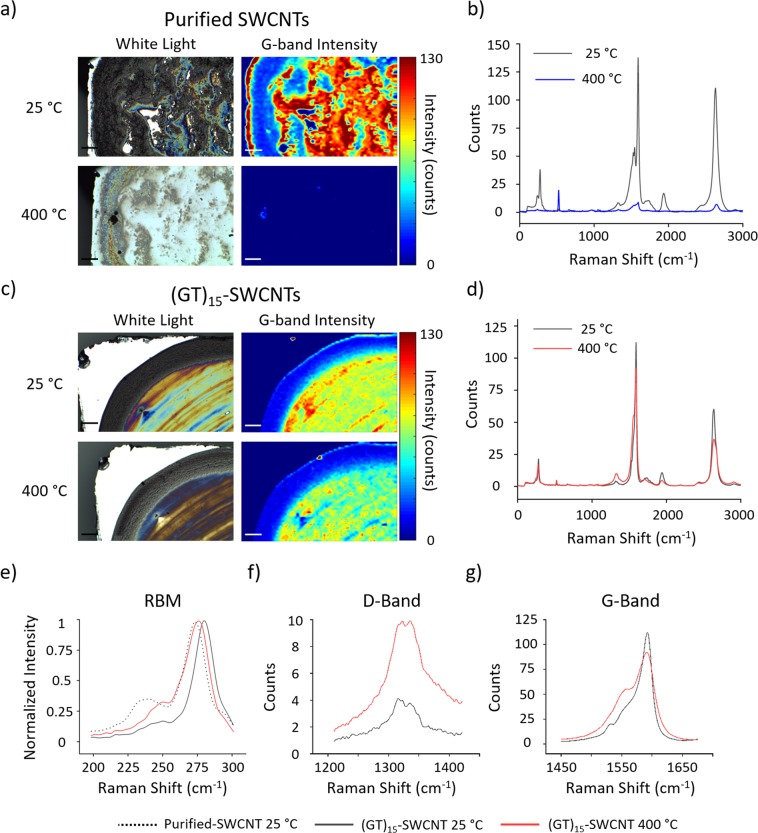
Brightfield images and G-band confocal Raman intensity maps of (**a**) purified SWCNTs and (**c**) (GT)_15_-SWCNTs dried on silicon wafers before and after a 30-minute exposure to 400 °C. The average spectra of SWCNT-containing pixels in confocal Raman area scans of (**a**,**c**) are shown in (**b**,**d**) respectively. Average Raman spectra zoomed-in to show detail in (**e**) normalized RBM, (**f**) D-band, and (**g**) G-band of (GT)_15_-SWCNTs at room temperature and after a 30-minute exposure to 400 °C. Scale Bar = 100 μm.

To confirm that the structural integrity of the SWCNTs is preserved after exposure to elevated temperatures, we performed confocal Raman microscopy on purified SWCNTs and (GT)_15_-SWCNTs dried on silicon wafers. Initially, both samples produced intense G-band signals at room temperature (Fig. 3a–c). However, a 30-minute exposure to 400 °C resulted in significantly reduced G-band intensity and a loss of other characteristic Raman features of purified SWCNTs (Fig. 3a,b), indicating that the SWCNTs were mostly decomposed or significantly damaged at the elevated temperature. Conversely, the G-band intensity maps of SWCNTs functionalized with DNA displayed minimal differences before and after exposure to 400 °C, although the slight decrease in intensity suggests that a small portion of SWCNTs had decomposed (Fig. 3c,d). As expected from TGA profiles, both samples were fully decomposed at 700 °C (Fig. S10), resulting in spectra containing a single peak corresponding to the silicon wafer (528 cm^−1^). Regarding the RBM spectral feature, (GT)_15_-SWCNTs at room temperature were shifted ~6 cm^−1^ from the RBM value of purified SWCNTs and did not have the same features below 240 cm^−1^ due to presence of a DNA-wrapping (Fig. 3e)^45–47^. Interestingly, exposure of (GT)_15_-SWCNTs to 400 °C induced a 5 cm^−1^ shift from its original position back to the value of the purified SWCNT signal. These data suggest that most of the DNA had decomposed, leaving behind SWCNTs that were structurally similar to the purified samples. Comparison of average Raman spectra before and after heating (GT)_15_-SWCNTs to 400 °C (Fig. 3d) showed an increase in the overall intensity of the D-band and a local increase at 1560 cm^−1^ of the G-band (Fig. 3f,g). These changes are characteristic of increasing amounts of amorphous carbon^48,49^, which when taken with the decreased DNA content observed from the RBM signals, confirm the formation of a protective char around the SWCNTs.

**Figure 4:**
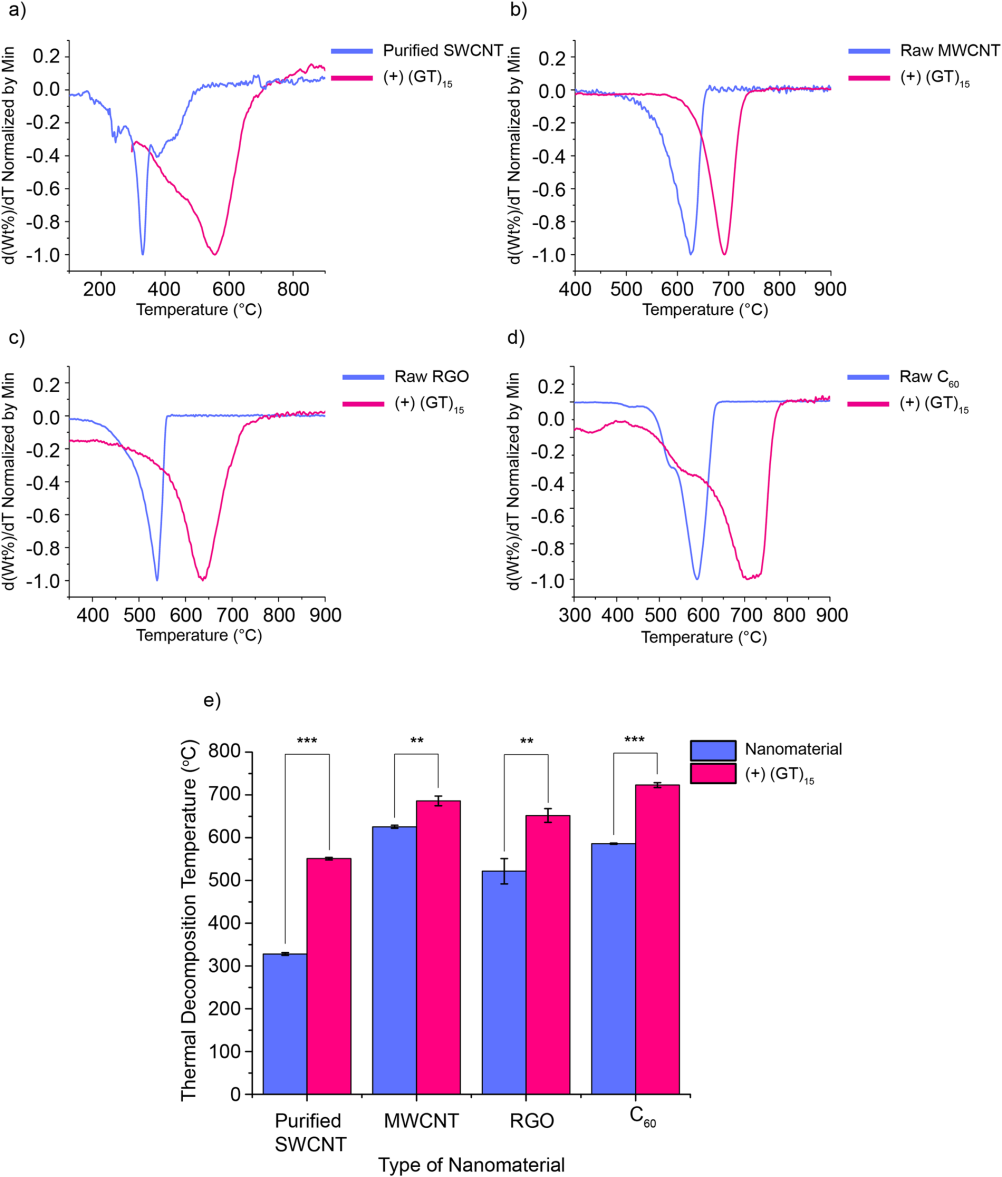
First-derivative weight percent versus temperature plots for (GT)_15_ DNA functionalized- (**a**) SWCNTs, (**b**) MWCNTs, (**c**) RGO, and (**d**) C_60_. (**e**) Bar graph to compare the increase in the thermal decomposition temperature of the carbon nanomaterials functionalized with DNA to that of raw/purified nanomaterials. TGA was repeated three times (n = 3) for each sample. Two-sample t-tests were performed (**P < 0.01, ***P < 0.001).

To demonstrate a potential flame-retardancy effect of DNA on other carbon nanomaterials, we functionalized MWCNTs, RGO and C_60_ with the same (GT)_15_ DNA sequence and quantified their thermal decomposition temperature (the temperature of the second significant mass loss in the first-derivative weight versus temperature profiles). Similar to SWCNTs, DNA wraps around MWCNTs and C_60_ by hydrophobic π-π stacking interactions^50,51^, while it randomly orients in between the RGO sheets through π-π stacking interactions^52,53^. In all these cases, DNA causes single particle exfoliation of these carbon nanomaterials in water. In a similar fashion, weight percent versus temperature and first derivative plots were created for all of the materials (Figs 4a–d and S11–S13). Comparing all DNA-carbon nanomaterials with the appropriate controls, the bar graph in Fig. 4e indicates substantial increases in the thermal decomposition temperature of all examined carbon nanomaterials in the order SWCNTs > C_60_ > RGO > MWCNTs.

**Figure 5:**
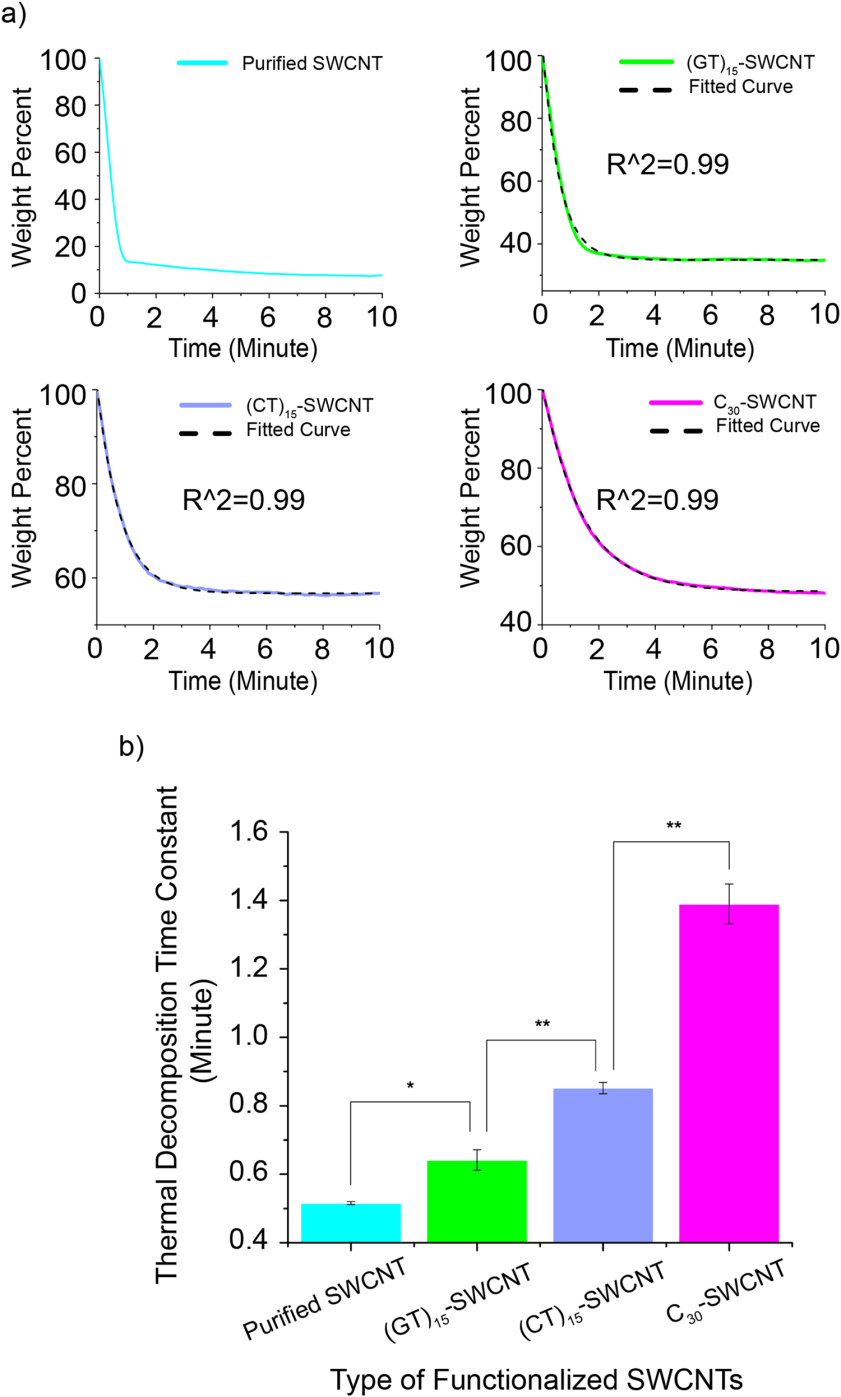
(**a**) Weight percent versus time profiles for purified SWCNTs, and DNA-SWCNTs composed of (GT)_15_, (CT)_15_, or C_30_ DNA sequences, when the temperature is instantaneously increased from 400 to 700 °C. The data are fitted to a single exponential decay and the resultant curve is plotted as a black dotted line. (**b**) Bar graph to compare the thermal decomposition rate (1/thermal decomposition time constant) of raw SWCNTs and DNA-SWCNTs made from (GT)_15_, (CT)_15_, or C_30_ DNA sequences. TGA was repeated three times (n = 3) for each sample. A two-sample t-test was performed (*p < 0.05, **p < 0.01).

In addition to the thermal decomposition temperature, the decomposition rate in an instantnous temperature increase is another important parameter associated with thermal stability. To compare the raw SWCNT thermal decomposition rate to that of the DNA-SWCNT hybrids, we performed a weight percent versus time assay when the material was subjected to a rapid temperature increase from 400 to 700 °C (Fig. 5). It is known that the compactness of the DNA corona on the SWCNTs can be modulated by DNA base type^54^. Therefore, in addition to the (GT)_15_ DNA sequence, we functionalized the SWCNTs with cytosine-containing sequences (CT)_15_ and C_30_, as cytosine-rich sequences display a significantly reduced desorption from the SWCNTs compared to other sequences and form more compact packing structures on the SWCNTs, resulting in more DNA coverage on the SWCNTs^54^. Here, our hypothesis is that more DNA bound to the SWCNT should correlate to slower thermal decomposition rates for DNA-SWCNT hybrids. Indeed, the increase in the cytosine content of the sequence significantly decreased the apparent decomposition rates (increased the decomposition time constants) of the resultant DNA-SWCNT hybrids (Fig. 5a,b). Figures S14 and S15 demonstrate the weight percent versus time profiles of (GT)_15_-, (CT)_15_-, and (C)_30_-SWCNT hybrids and their appropriate controls. The temperature was first set to 400 °C and held constant for 30 minutes to allow for DNA char formation. After 30 minutes, the temperature was rapidly raised to 700 °C and kept constant for 30 minutes. Figure S14b–d demonstrate that there is no significant mass loss after the first rapid temperature increase where the temperature is held constant at 400 °C for 30 minutes. This confirms the DNA char assisted protection for SWCNTs against decomposition at 400 °C. We fitted the decay processes of DNA-SWCNTs in the second step (rapid temperature increase from 400 to 700 °C) to a single exponential (Figs 5a and S16) and report a quantified time constant for each sample (Table S1). Due to the poor fit of the data in the case of purified SWCNTs (Fig. S16a), which may be attributed to limitations in the instrument’s ability to instantaneously raise the temperature to the desired set-point, the time constant for this sample was calculated from its definition (i.e. the time required for a system to reach 1/e (~36.8%) of its initial value). Comparing all DNA sequences to the SWCNT control, Fig. 5b shows that DNA-SWCNTs decompose in the rate order of C_30_ < (CT)_15_ < (GT)_15_. Therefore, an increase in the relative cytosine content in the DNA sequence significantly enhances the thermal stability characteristics of the SWCNTs.

## Conclusions

**Figure 6:**
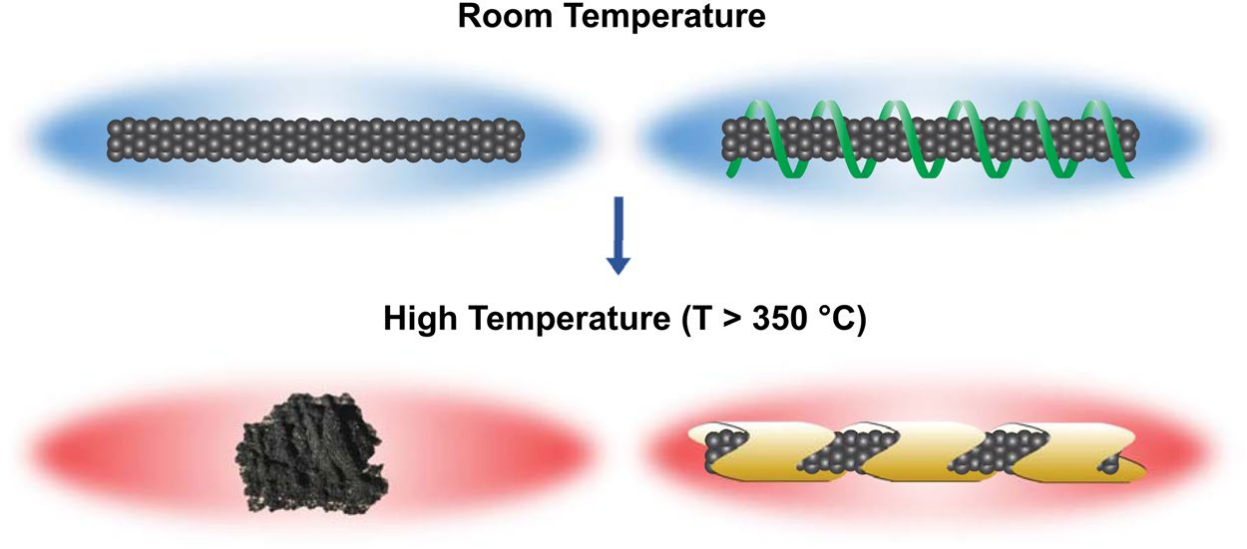
Illustration of DNA char assisted protection for SWCNTs against thermal decomposition. SWCNTs without DNA coverage decompose at high temperatures while SWCNTs noncovalently wrapped with DNA remain stable at high temperatures.

In this work, we examined the thermal decomposition temperature and rate of DNA functionalized carbon nanomaterials using thermogravimetric analysis. We find that functionalization with DNA significantly increases the thermal decomposition temperature of all examined carbon nanomaterials in the order SWCNTs > C_60_ > RGO > MWCNTs. We attribute these increases in decomposition temperature to the fact that DNA, an intumescent molecular entity that increases in volume upon heating, shields the hybridized carbon nanomaterials from flame and elevated temperatures (Fig. 6). Moreover, we control the decomposition rate of the SWCNTs by manipulating the sequence of the DNA and show that a higher cytosine content corresponds to a higher degree of flame retardancy. Enhancing the thermal decomposition properties of carbon nanomaterials with non-toxic flame retardants enables the realization of environmentally friendly high temperature sensing and electronics applications.

## Materials and Methods

### DNA assisted dispersion of SWCNTs

SWCNTs produced by the HiPco process (Nanointegris) were used throughout the study. SWCNTs were dispersed with single-stranded DNA oligonucleotides in 1 mL of 100 mM NaCl (Sigma-Aldrich), by adding 1 mg of raw nanotubes to 2 mg of desalted (GT)_n_ (n = 3, 6, 9, 12, 15, or 30), (CT)_15_, or C_30_ oligonucleotide (Integrated DNA Technologies) in a microcentrifuge tube. The mixtures were ultrasonicated using a 1/8” tapered microtip (Sonics Vibracell; Sonics & Materials) for 30 minutes at 40% amplitude, with an average power output of 8 W, in a 0 °C temperature-controlled microcentrifuge tube holder. After sonication, the dispersion was ultracentrifuged (Sorvall Discovery M120 SE) for 30 minutes at 250,000 g, and the top 80% of the supernatant was extracted. The concentration was determined with a UV/vis/NIR spectrophotometer (Jasco, Tokyo, Japan), using the extinction coefficient of A_910_ = 0.02554^55^ L.mg^−1^.cm^−1^. The free DNA was removed using 100 kDa Amicon centrifuge filters (Millipore). For each sample, filtration was repeated 4 times, and the DNA-SWCNTs pellet was resuspended in DI water in each step including the final step.

### Preparation of the purified SWCNTs from the (GT)_15_-SWCNTs

The free DNA was first removed following the protocol reported in the previous section. The DNA bound to the SWCNTs was displaced with a surfactant (sodium deoxycholate (SDC)) according to the procedure reported in our previous study^56^. The SDC in the resultant SDC-SWCNT samples was displaced and removed using multiple Amicon filtration steps (5–8 times). The purified SWCNT aggregates were then collected for further analysis.

### DNA assisted dispersion of MWCNTs, RGO, and C_60_

MWCNTs (Alfa Aesar), RGO (Graphene Supermarket), and C_60_ (Alfa Aesar) were dispersed with (GT)_15_ DNA sequence in 1 mL of 100 mM NaCl, by adding 1 mg of raw nanomaterials to 2 mg of desalted DNA in a microcentrifuge tube. The mixtures were ultrasonicated using a 1/8” tapered microtip for 30 minutes (Only the C_60_ solution was ultrasonicated for 90 minutes) at 40% amplitude, with an average power output of 8 W, in a 0 °C temperature-controlled microcentrifuge tube holder. The free DNA was removed using 100 kDa Amicon centrifuge filters (Millipore), and the DNA functionalized carbon nanomaterials pellet was resuspended in DI water.

### Thermogravimetric analysis (TGA)

500 µL of samples of pure desalted (GT)_n_ DNA, raw/purified carbon nanomaterials, or DNA functionalized carbon nanomaterials with free DNA and salt removed were added to Platinum pans and the water of the samples evaporated. The pans were placed in a TGA instrument (TA Instruments, Q500) and the instrument was programmed to perform different functions. To quantify the thermal decomposition temperatures of the samples, the temperature was increased by 20 °C/min from room temperature to 1000 °C, through a ramp function. The (GT)_15_-SWCNT sample was additionally heated at a rate of 10 °C/min to compare the effect of heating rate on the thermal decomposition temperature. To investigate the thermal decomposition rates (time constants) of the DNA-SWCNTs, the temperature was jumped to 400 °C and then 700 °C, and held constant at each of the temperatures for 30 minutes to make sure that the DNA decomposes at the first step and SWCNTs decompose at the second step.

### Transmission electron microscopy (TEM)

(GT)_15_-SWCNT samples (free DNA and salt removed by filtration) at room temperature were imaged on carbon-coated TEM grids (Electron Microscopy Sciences) by depositing a 5 μL drop on the center of the grid, and letting it dry in room temperature. Additionally, to perform the TEM of (GT)_15_-SWCNT samples decomposed at 400 °C and 700 °C, a 5 μL drop of the sample (free DNA and salt removed by filtration) was placed on the center of PELCO Silicon Nitride Support grids (Ted Pella), and dried at room temperature. The grids were then placed in TGA pans and the temperature was jumped to either 400 °C or 700 °C, and held constant at each temperature for 30 minutes. The grids were then used for TEM imaging (JEOL JEM-2100F) operating at 200 kV.

### Confocal raman microscopy

Purified SWCNTs or (GT)_15_-SWCNTs (free DNA and salt removed by filtration) were deposited on silicon carbide wafers and the water was evaporated. The samples were imaged using a WITec alpha300 R confocal Raman microscope with a 532 nm excitation laser and 10x objective, and Raman spectra were obtained in 10 µm intervals across the image area. Each sample was imaged directly after drying and again after incubation at either 400 °C or 700 °C for 30 minutes. The SWCNT-containing Raman spectra were determined by the presence of the G-band, and all SWCNT-containing pixels were averaged to obtain a single Raman spectrum for each condition.

### Statistical analysis

Statistical measurements and analyses were performed in OriginPro 2016 using two-sample t-tests under the null hypothesis.

## Supplementary information

Supporting Information
