# Supplementary material for: Enhancing the Thermal Stability of Carbon Nanomaterials with DNA
Source: Sci Rep. 2019 Aug 15;9:11926. doi: 10.1038/s41598-019-48449-x (PMC6695385; doi:10.1038/s41598-019-48449-x)
Supplement: Supplementary file 1 — Supporting Information [file 41598_2019_48449_MOESM1_ESM.pdf]

Supporting Information

Enhancing the Thermal Stability of Carbon Nanomaterials with DNA

Mohammad Moein Safaee[1], *Mitchell Gravely*[1], *Adeline Lamothe*[1], *Megan McSweeney*[1], *Daniel Roxbury*[1*]

[1]*Department of Chemical Engineering, University of Rhode Island, Kingston, Rhode Island 02881, United States*

Corresponding Author

*E-mail: roxbury@uri.edu.

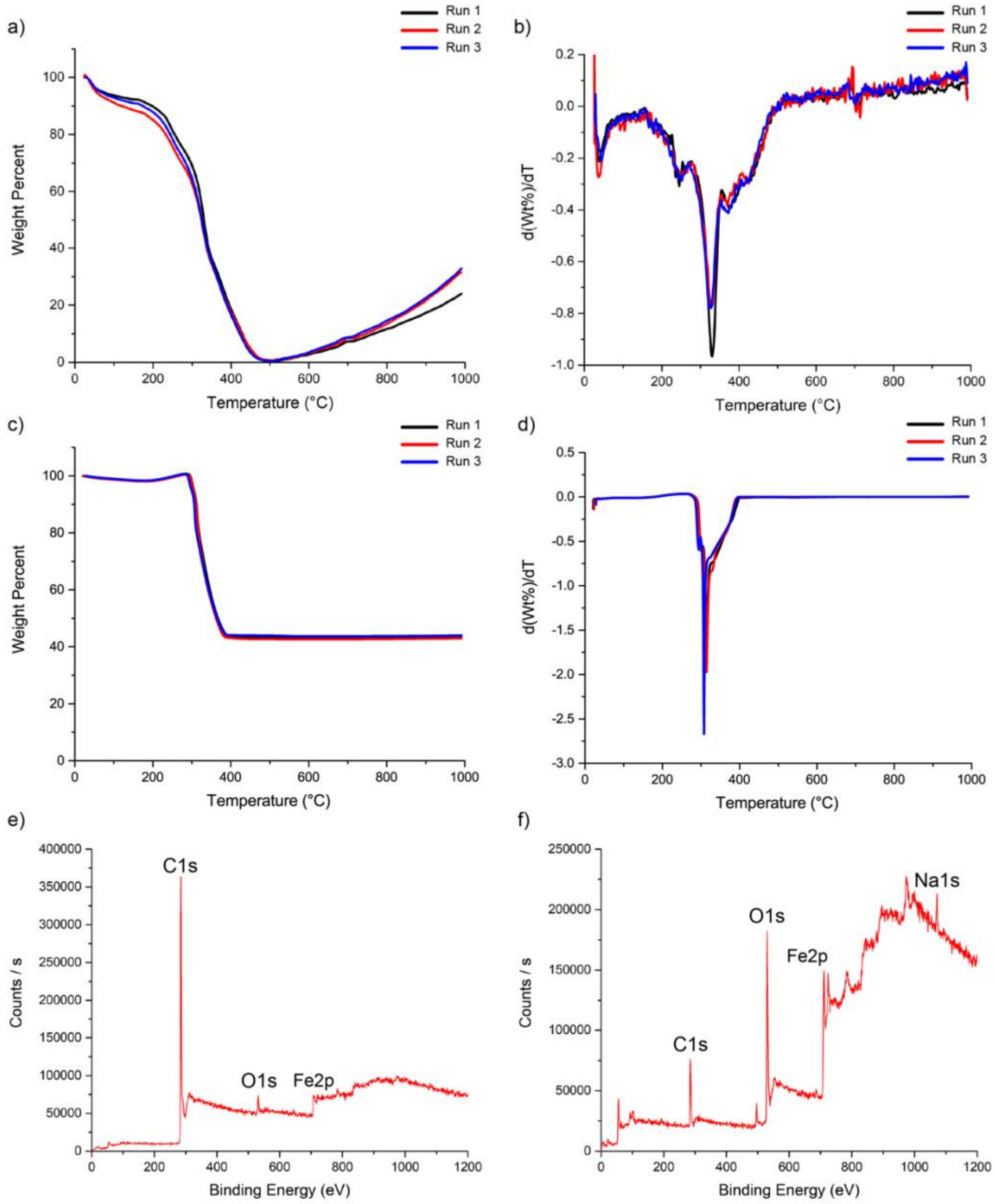

Name	Atomic %
Carbon (as C1s)	93.02
Oxygen (as O1s)	4.97
Iron (as Fe2p)	2.01

Name	Atomic %
Oxygen (as O1s)	45.81
Iron (as Fe2p)	13.69
Carbon (as C1s)	35.61
Sodium (as Na1s)	4.89

Figure S1. (a) Weight percent versus temperature profiles of purified SWCNTs. (b) First-derivative weight percent versus temperature profiles of purified SWCNTs. (c) Weight percent versus temperature profiles of raw HiPco SWCNTs. (d) First-derivative weight percent versus temperature profiles of raw HiPco SWCNTs. XPS data and the corresponding atomic percentages of the components of (e) raw HiPco SWCNTs at room temperature, and (f) residual material from raw HiPco SWCNTs elevated to 700 °C for 30 minutes.

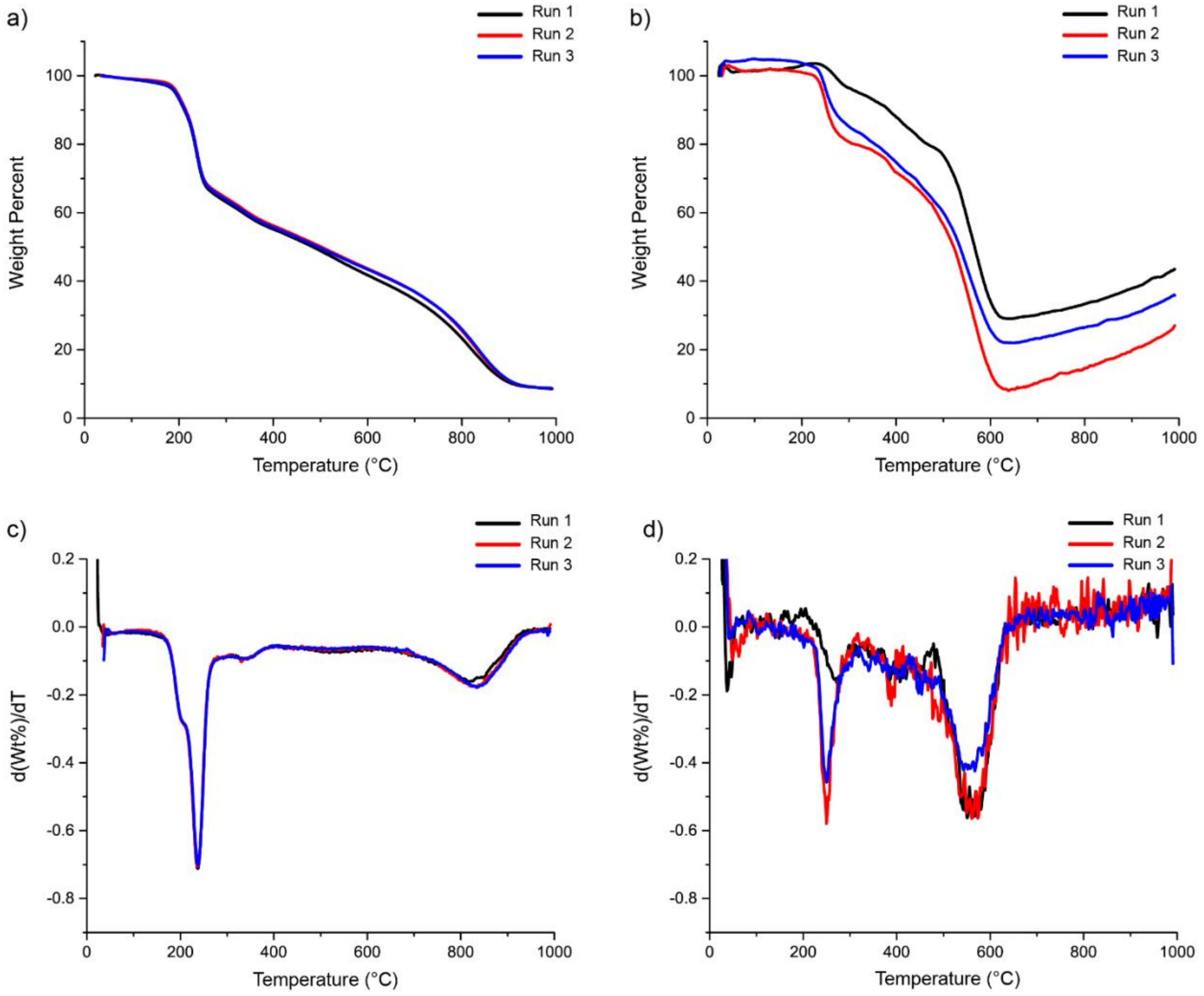

Figure S2. Weight percent versus temperature profiles of (a) $(GT)_6$ DNA sequence, and (b) $(GT)_6$-SWCNTs. First-derivative weight percent versus temperature profiles of (c) $(GT)_6$ DNA sequence, and (d) $(GT)_6$-SWCNTs.

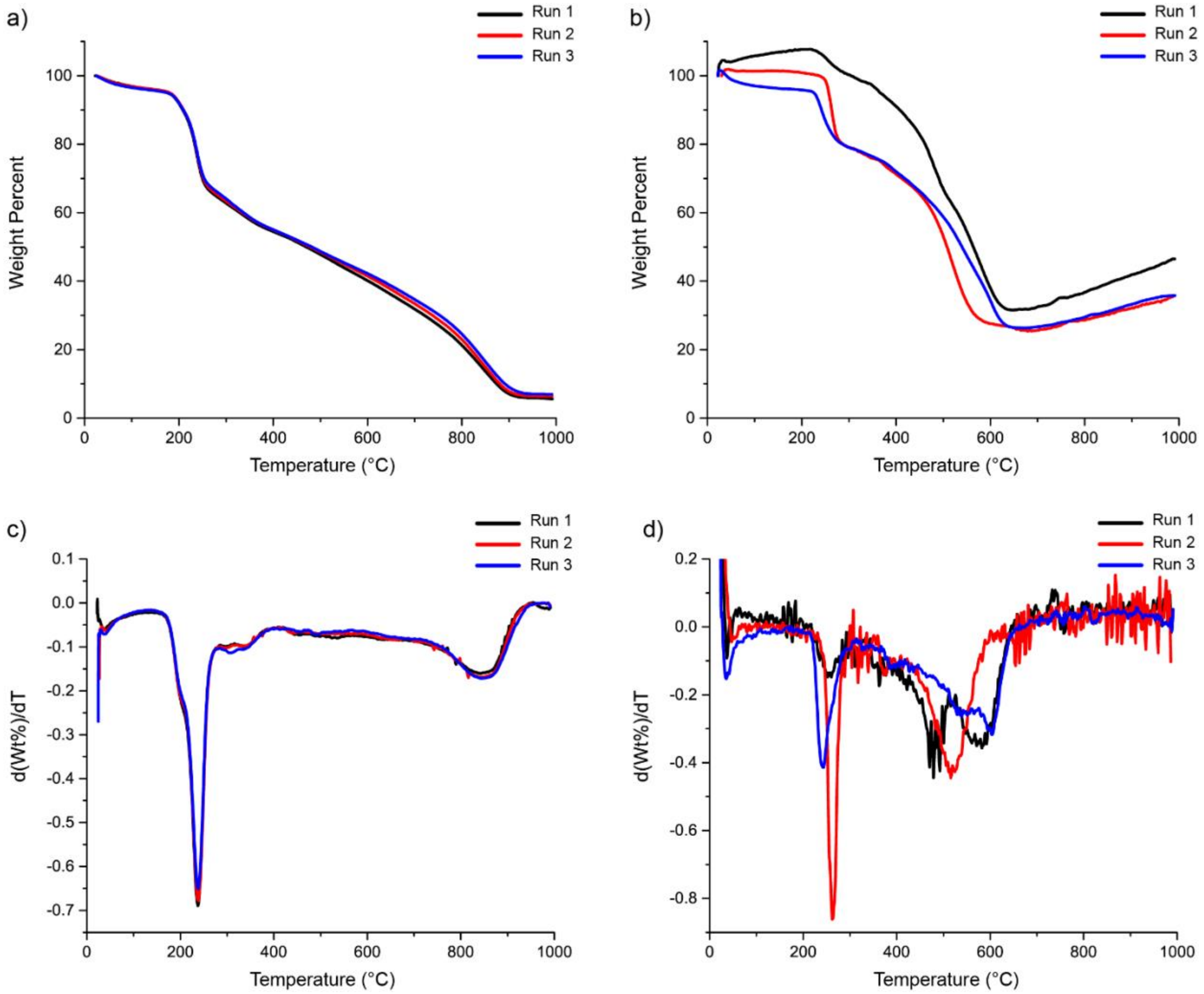

Figure S3. Weight percent versus temperature profiles of (a) $(GT)_9$ DNA sequence, and (b) $(GT)_9$-SWCNTs. First-derivative weight percent versus temperature profiles of (c) $(GT)_9$ DNA sequence, and (d) $(GT)_9$-SWCNTs.

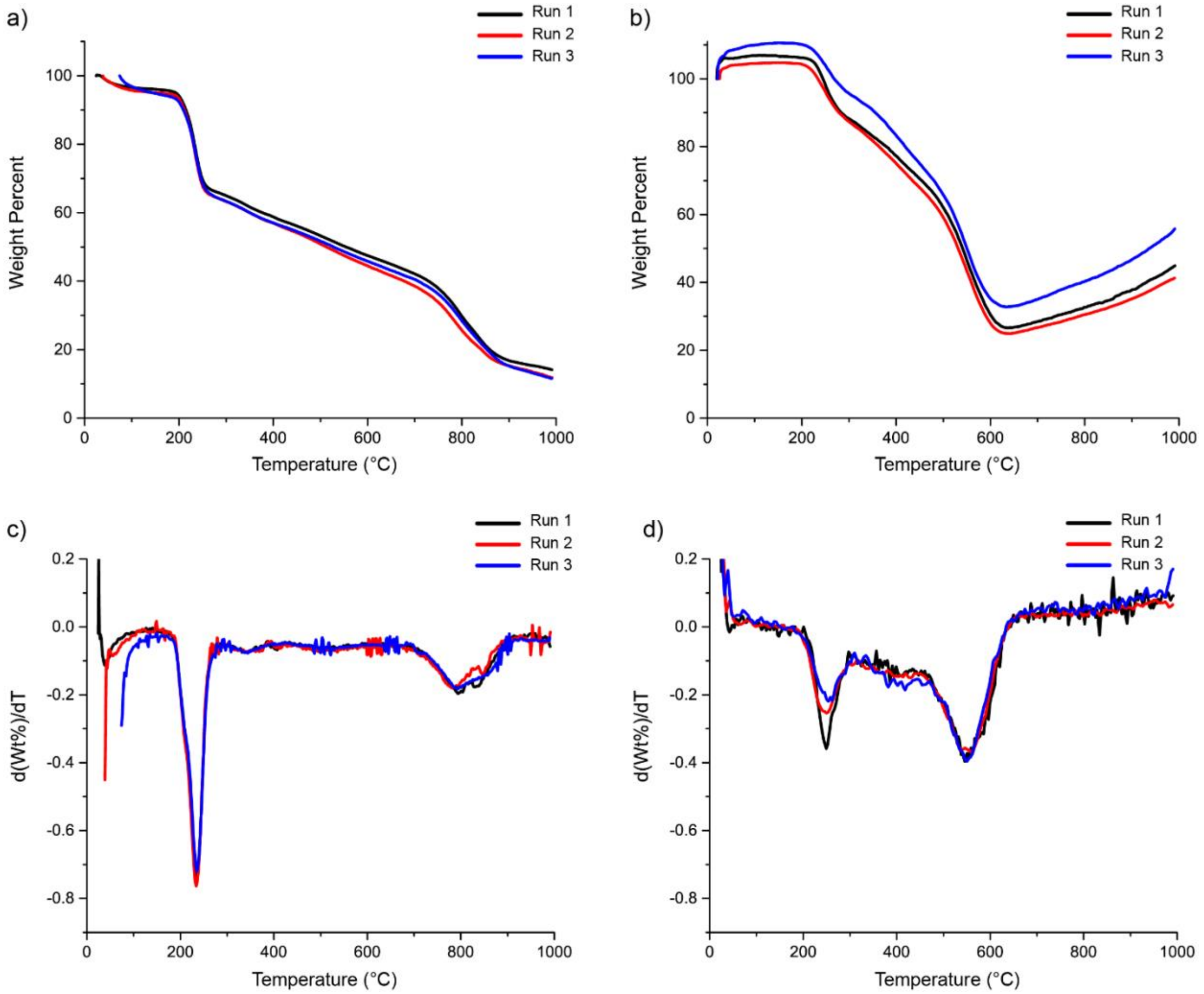

Figure S4. Weight percent versus temperature profiles of (a) $(GT)_{12}$ DNA sequence, and (b) $(GT)_{12}$-SWCNTs. First-derivative weight percent versus temperature profiles of (c) $(GT)_{12}$ DNA sequence, and (d) $(GT)_{12}$-SWCNTs.

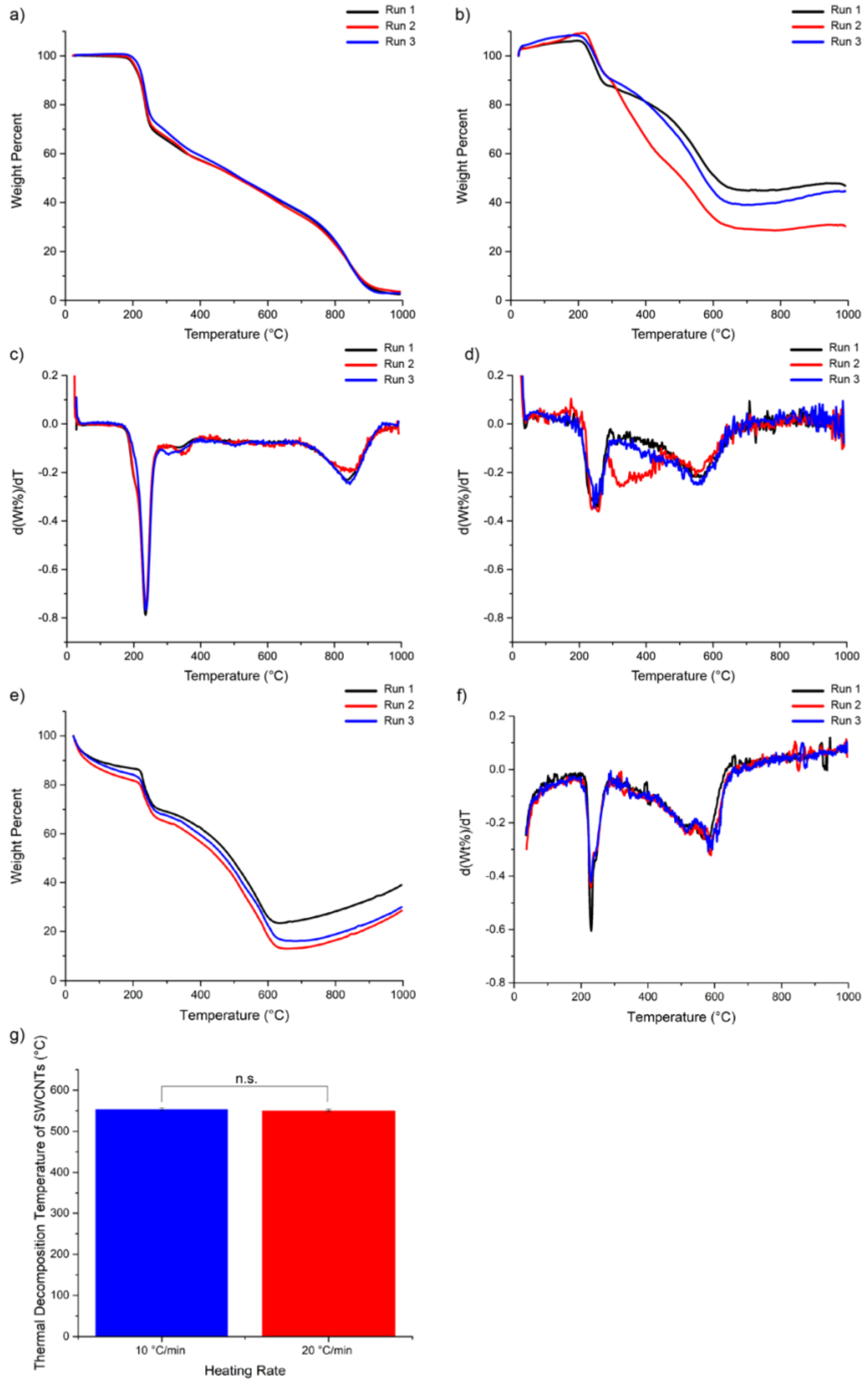

Figure S5. Weight percent versus temperature profiles of (a) $(GT)_{15}$ DNA sequence, and (b) $(GT)_{15}$-SWCNTs. First-derivative weight percent versus temperature profiles of (c) $(GT)_{15}$ DNA sequence, and (d) $(GT)_{15}$-SWCNTs. (e) Weight percent versus temperature profiles of $(GT)_{15}$-SWCNTs at the heating rate of 10 °C/min. (f) First-derivative weight percent versus temperature profiles of $(GT)_{15}$-SWCNTs at the heating rate of 10 °C/min. (g) The temperature of the second peak (thermal decomposition temperature of SWCNTs) in the first-derivative weight percent versus temperature profiles of $(GT)_{15}$-SWCNTs. TGA was repeated three times (n=3) for each sample. A two-sample t-test was performed (n.s., $P > 0.05$).

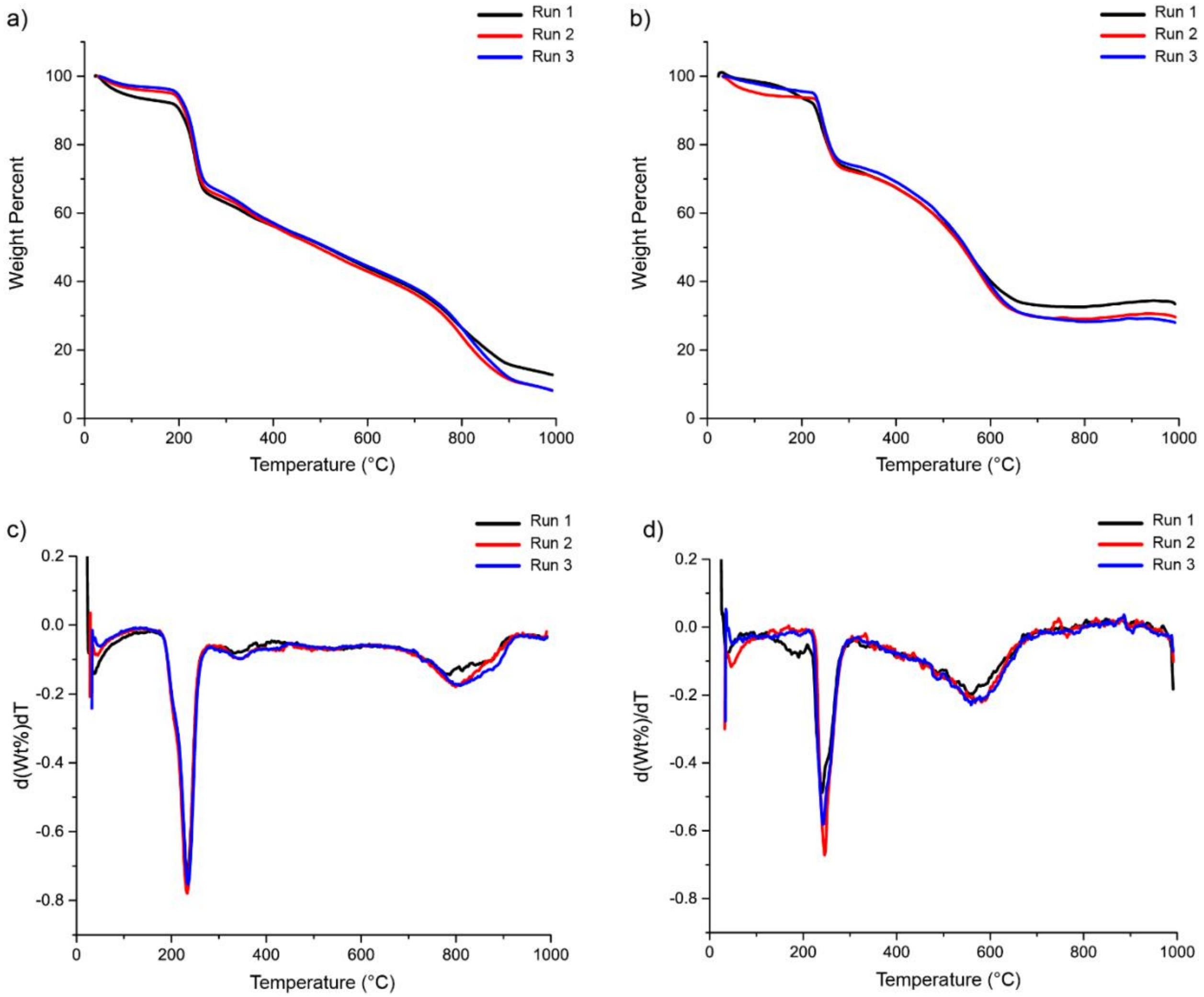

Figure S6. Weight percent versus temperature profiles of (a) $(GT)_{30}$ DNA sequence, and (b) $(GT)_{30}$-SWCNTs. First-derivative weight percent versus temperature profiles of (c) $(GT)_{30}$ DNA sequence, and (d) $(GT)_{30}$-SWCNTs.

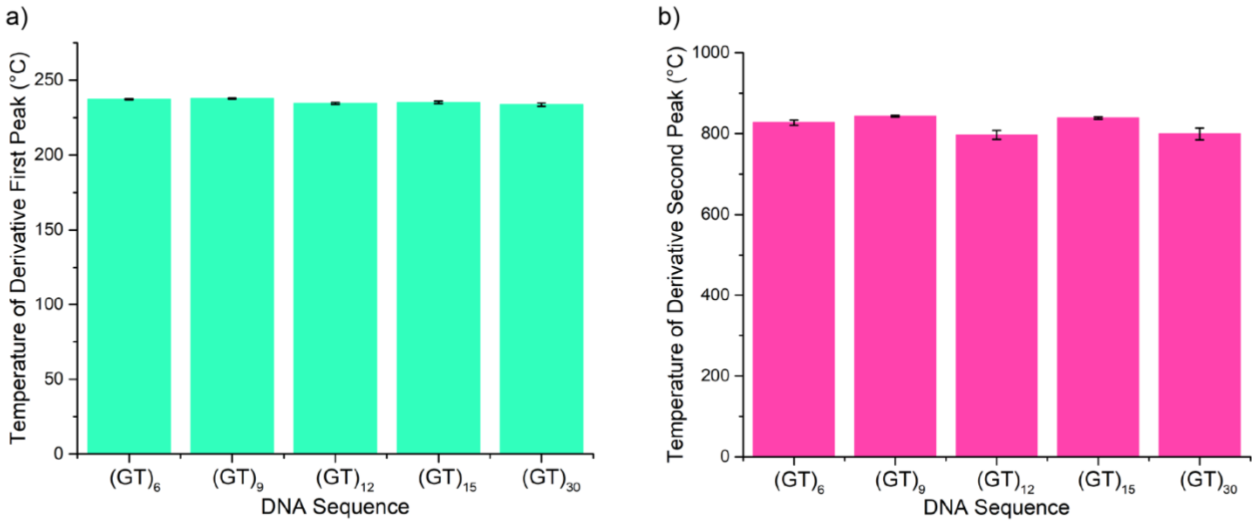

Figure S7. The temperature of (a) the first peak, and (b) the second peak, in the first-derivative weight versus temperature profiles of $(GT)_n$ DNA sequences (n: 6, 9, 12, 15, 30). TGA was repeated three times (n=3) for each sample. A two-sample t-test was performed between every two DNA sequences to compare the first and second peak temperatures. There was no significant difference ($P > 0.05$) in all comparisons.

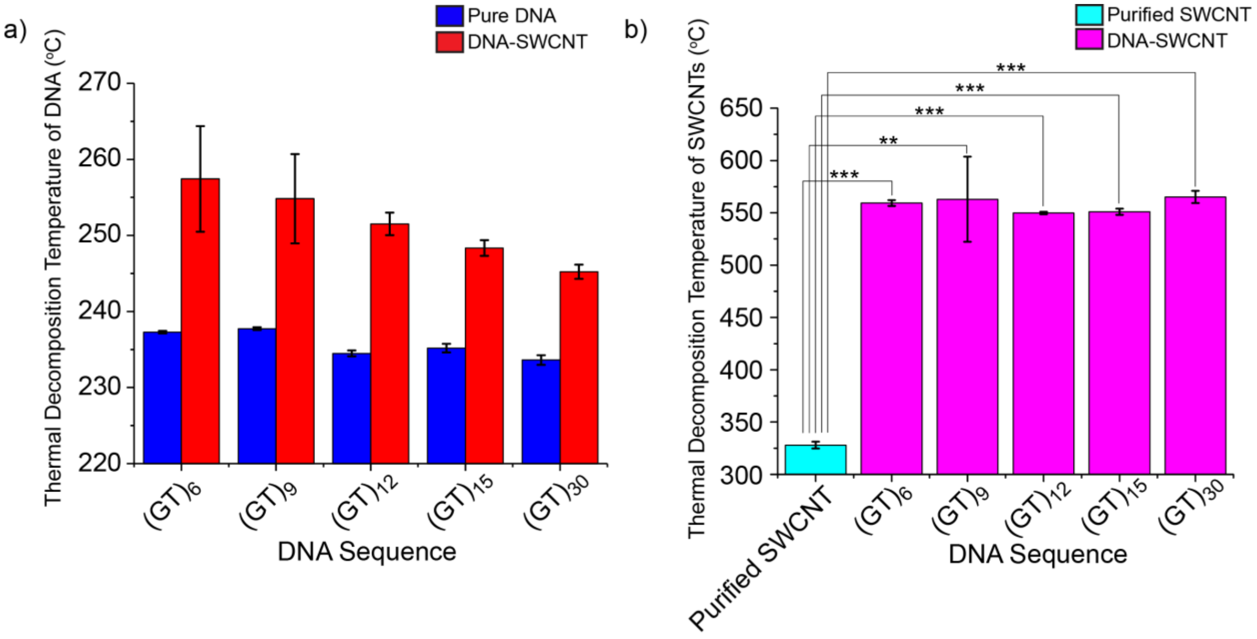

Figure S8. (a) The temperature of the first peak (thermal decomposition temperature of DNA) in the first-derivative weight versus temperature profiles of $(GT)_n$ DNA sequences (n: 6, 9, 12, 15, 30), and $(GT)_n$-SWCNT hybrids. The bar graph shows an increase in the thermal decomposition temperature of DNA in $(GT)_n$-SWCNT hybrids compared to that of $(GT)_n$ DNA sequences. This increase is not significant ($P > 0.05$) for all of the $(GT)_n$ DNA sequences. (b) The temperature of the second peak (thermal decomposition temperature of SWCNTs) in the first-derivative weight versus temperature profiles of purified SWCNTs and $(GT)_n$-SWCNT hybrids. The bar graph indicates a significant increase in thermal decomposition temperature of SWCNTs in $(GT)_n$-SWCNT hybrids compared to that of purified SWCNTs. TGA was repeated three times (n=3) for each sample. A two-sample t-test was performed (**, $P < 0.01$, ***, $P < 0.001$).

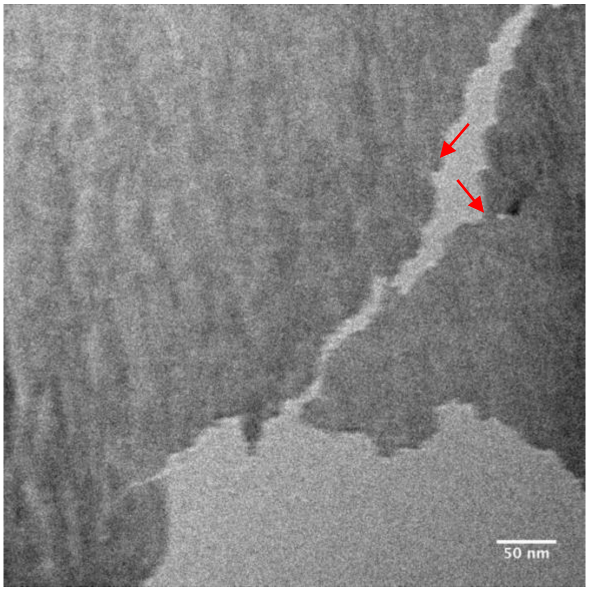

Figure S9. TEM image of $(GT)_{15}$ DNA sequence held at 400 °C for 30 minutes, demonstrating the char formed from DNA. A 10 mg/mL DNA solution was dried on a silicon nitride support grid. The temperature was rapidly increased to 400 °C and held constant for 30 minutes.

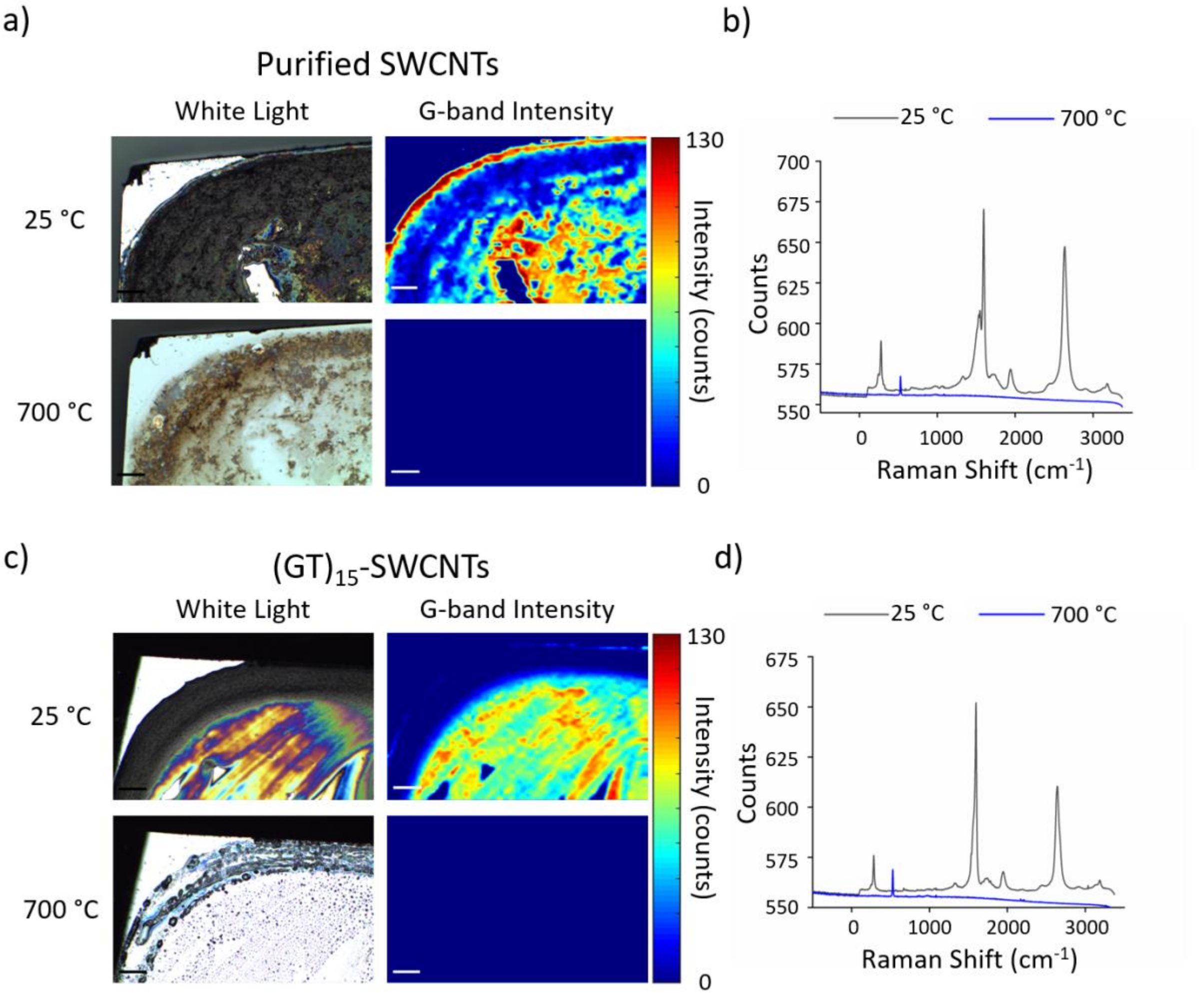

Figure S10. Brightfield images and G-band intensity maps of (a) purified SWCNTs and (c) $(GT)_{15}$-SWCNTs dried on silicon wafers before and after 30-minute exposure to 700°C. Average spectra of SWCNT-containing pixels in confocal Raman area scans of (a) and (c) are shown in (b) and (d) respectively. Scale Bar = 100 μm.

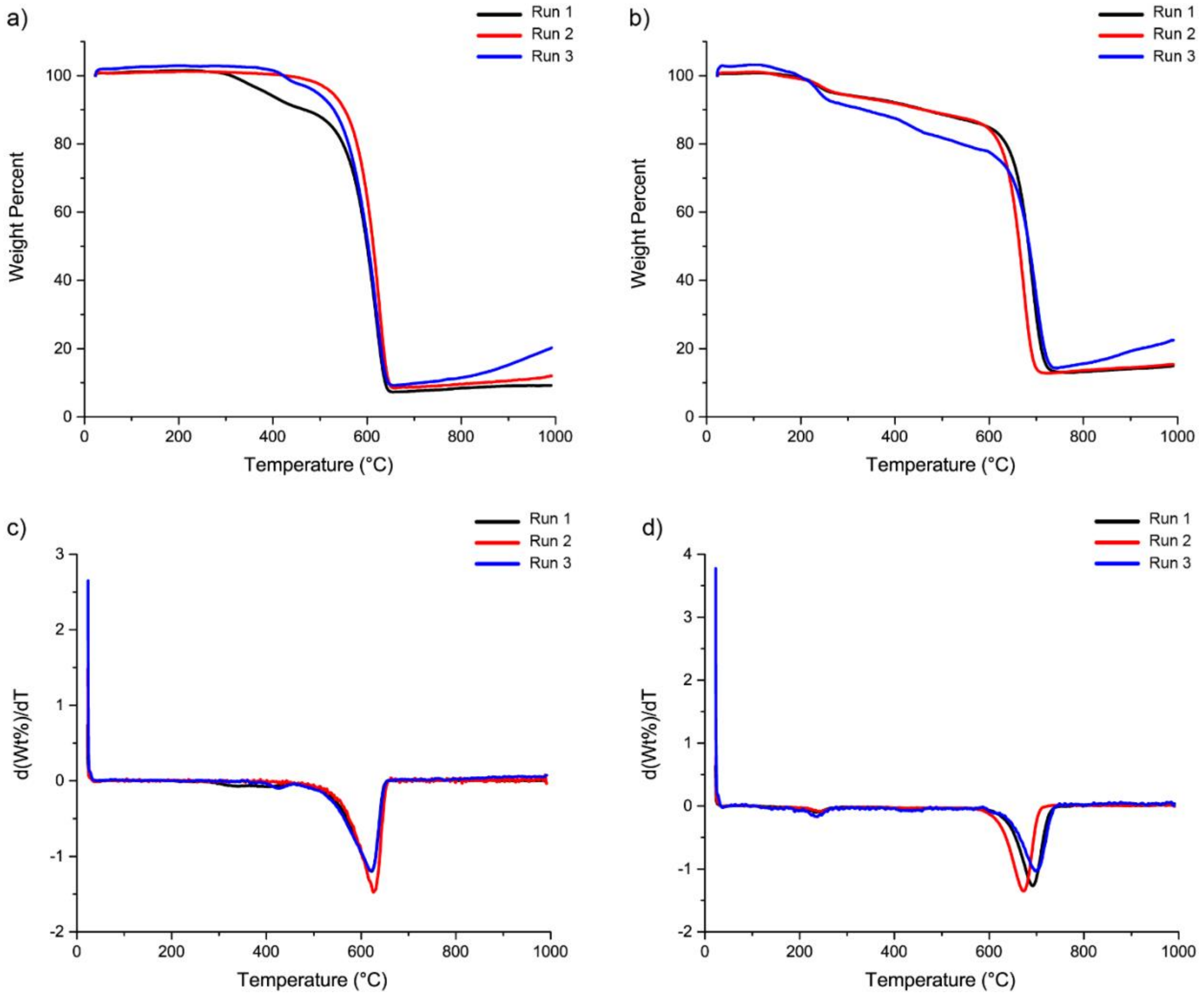

Figure S11. Weight percent versus temperature profiles of (a) raw MWCNTs, and (b) $(GT)_{15}$-MWCNTs. First-derivative weight percent versus temperature profiles of (c) raw MWCNTs, and (b) $(GT)_{15}$-MWCNTs.

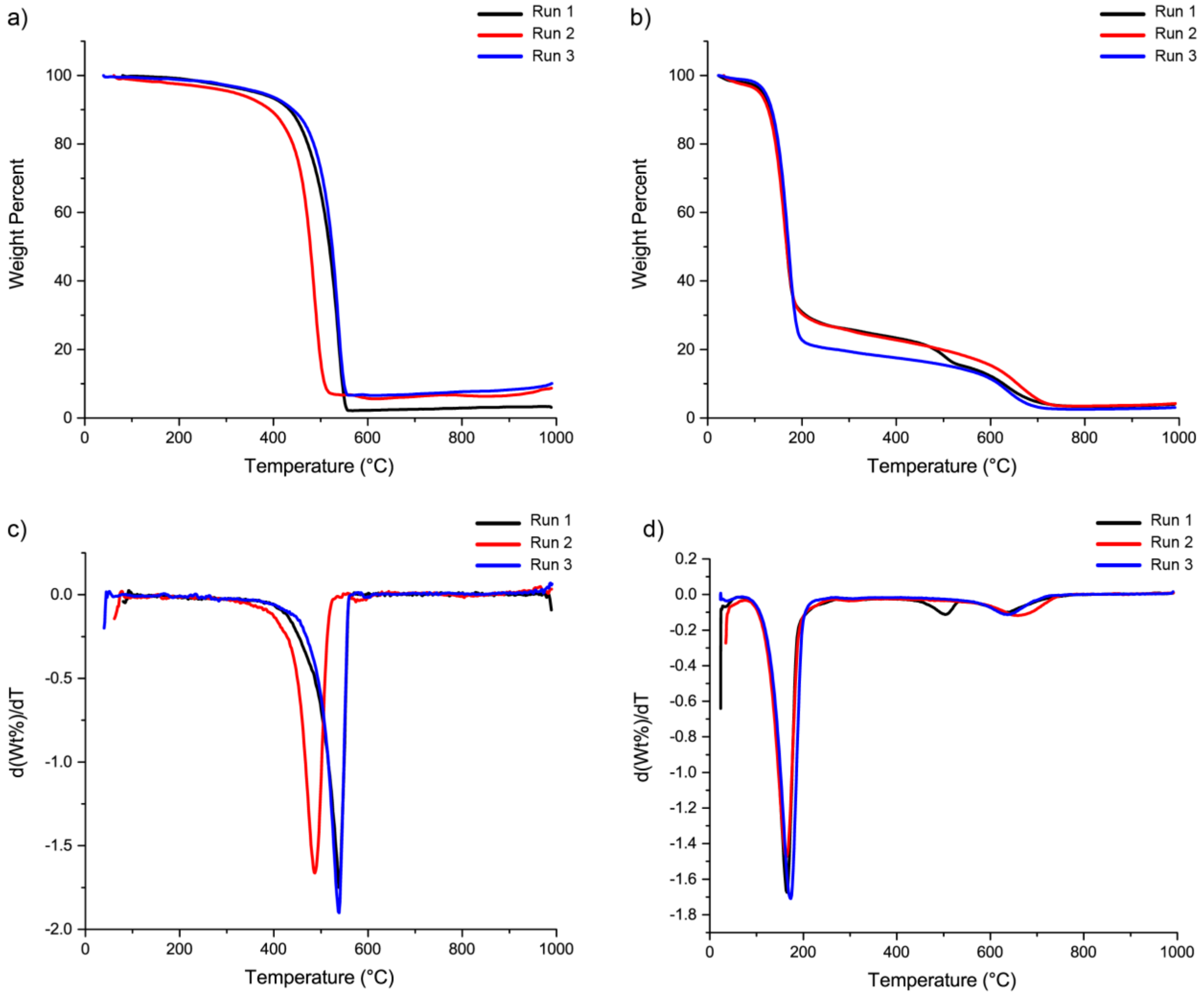

Figure S12. Weight percent versus temperature profiles of (a) raw RGO, and (b) $(GT)_{15}$-RGO. First-derivative weight percent versus temperature profiles of (c) raw RGO, and (b) $(GT)_{15}$-RGO.

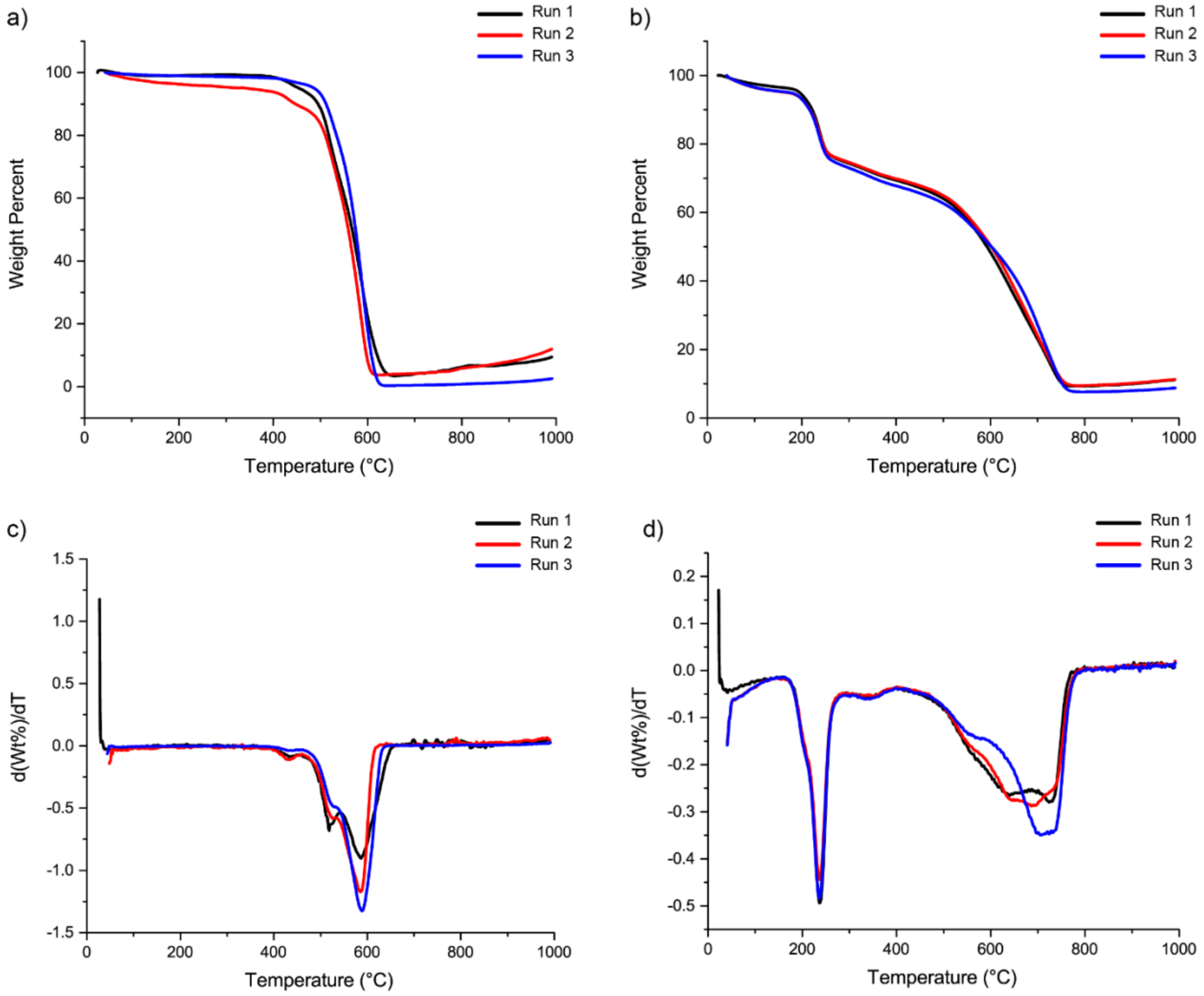

Figure S13. Weight percent versus temperature profiles of (a) raw C_{60}, and (b) $(GT)_{15}$-C_{60}. First-derivative weight percent versus temperature profiles of (c) raw C_{60}, and (b) $(GT)_{15}$-C_{60}.

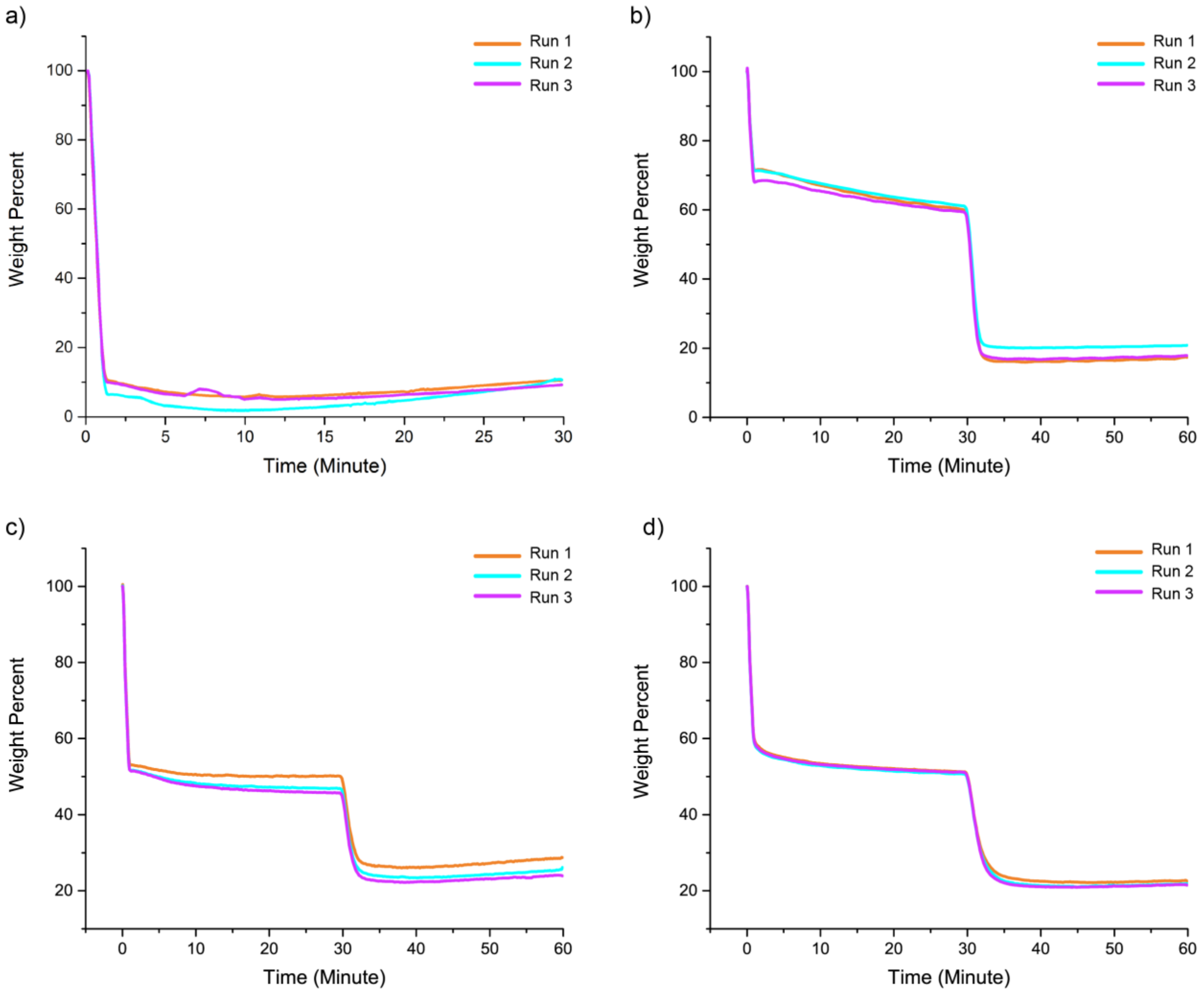

Figure S14. Weight percent versus time profiles of (a) purified SWCNTs, (b) $(GT)_{15}$-SWCNTs, (c) $(CT)_{15}$- SWCNTs, and (d) C_{30}- SWCNTs. The temperature was rapidly increased to 400 and 700 °C, respectively, and held constant at each temperature for 30 minutes.

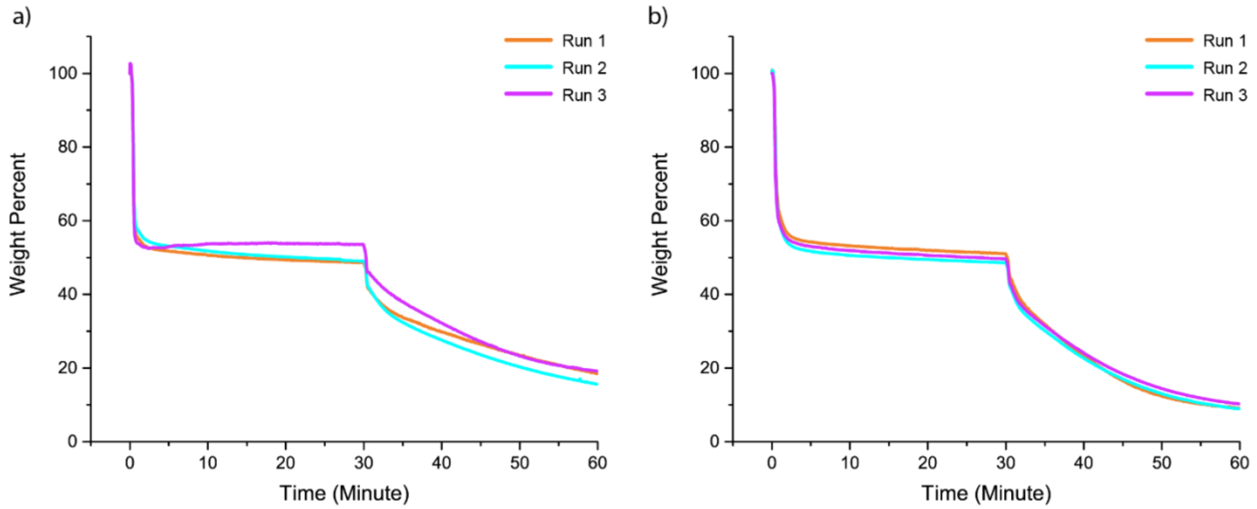

Figure S15. Weight percent versus time profiles of (a) $(GT)_{15}$ DNA sequence, and (b) C_{30} DNA sequence. The temperature was rapidly increased to 400 °C and then 700 °C, respectively, and held constant at each temperature for 30 minutes.

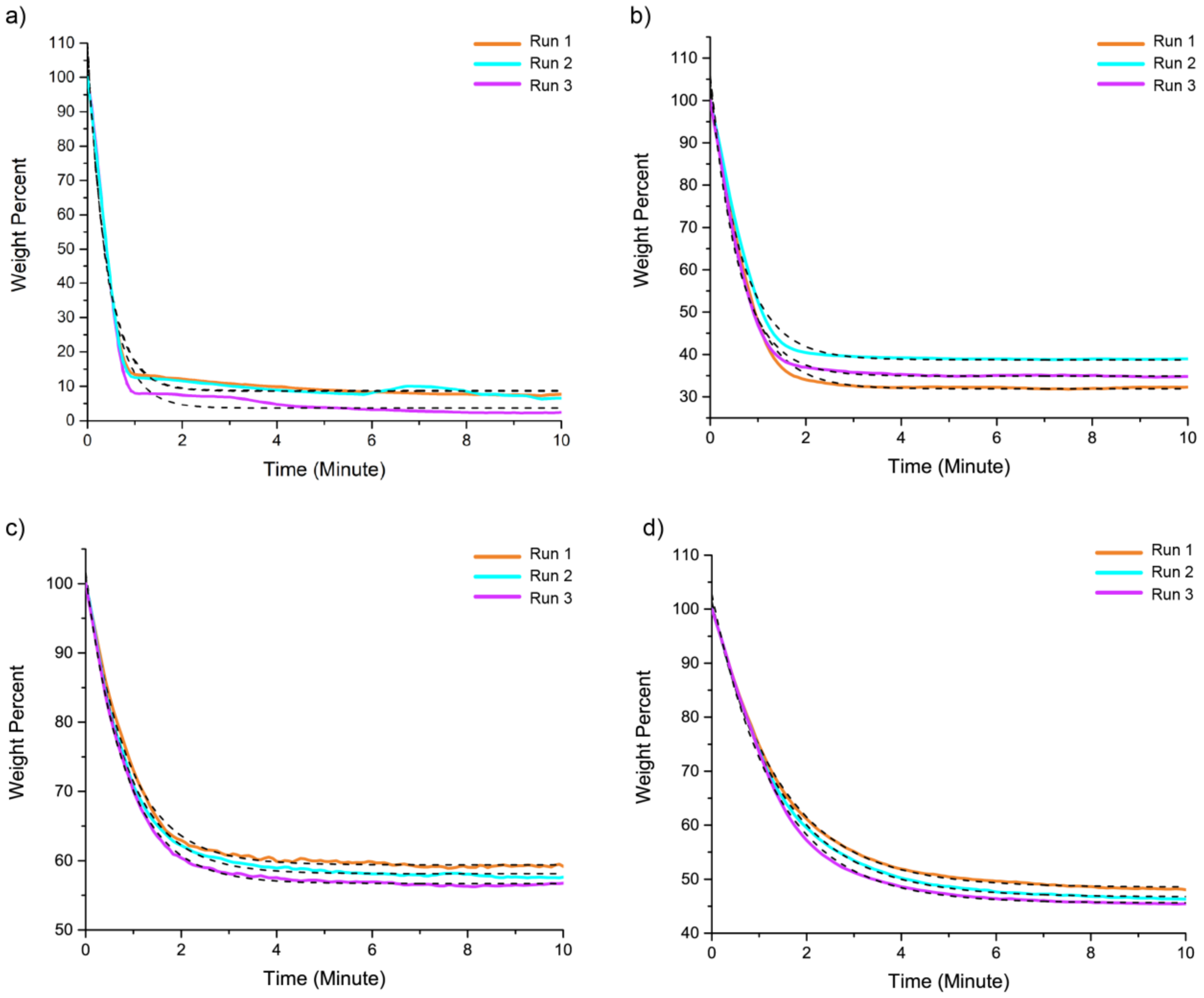

Figure S16. The first 10 minutes at the second step (where the temperature is rapidly increased from 400 °C to 700 °C) in the weight versus time profiles of (a) purified SWCNTs, (b) $(GT)_{15}$-SWCNTs, (c) $(CT)_{15}$-SWCNTs, and (d) C_{30}-SWCNTs. The decay processes were fitted to a single exponential function to extract the thermal decomposition rate (1/thermal decomposition time constant) of SWCNTs in DNA-SWCNT hybrids, in an instantaneous temperature increase procedure.

Table S1. Parameters extracted from single exponential fittings

$$y = y_0 + Ae^{-x/t}$$

Sample	y_0	A	t	R^2
	(°C)	Minutes	Minutes	
$(GT)_{15}$-SWCNT	35.14899± 3.440814	70.03354± 3.623106	0.64172± 0.030019	0.99
$(CT)_{15}$-SWCNT	58.07047± 1.352716	43.13829± 1.190044	0.85189± 0.016541	0.99
C_{30}-SWCNT	46.95515± 1.471978	54.79743± 2.309699	1.38979± 0.057981	0.99
